# Built to last? Barriers and facilitators of healthcare program sustainability: a systematic integrative review

**DOI:** 10.1186/s13012-023-01315-x

**Published:** 2023-11-13

**Authors:** Yvonne Zurynski, Kristiana Ludlow, Luke Testa, Hanna Augustsson, Jessica Herkes-Deane, Karen Hutchinson, Gina Lamprell, Elise McPherson, Ann Carrigan, Louise A. Ellis, Putu Novi Arfirsta Dharmayani, Carolynn L. Smith, Lieke Richardson, Genevieve Dammery, Nehal Singh, Jeffrey Braithwaite

**Affiliations:** 1 Centre for Healthcare Resilience and Implementation Science, Australian Institute of Health Innovation, Macquarie University, North Ryde, Australia Level 6, 75 Talavera Rd, NSW 2109; 2 NHMRC Partnership Centre for Health System Sustainability, Australian Institute of Health Innovation, Macquarie University, North Ryde, Australia; 3 Centre for Health Services Research, The University of Queensland, Brisbane, Australia; 4 Procome Research Group, Medical Management Centre, Department of Learning, Informatics, Management and Ethics, Karolinska Institutet, Solna, Sweden

**Keywords:** Sustainability, Healthcare systems improvement, Interventions, Complex systems, Systematic review

## Abstract

**Objective:**

To identify barriers and facilitators associated with the sustainability of implemented and evaluated improvement programs in healthcare delivery systems.

**Data sources and study setting:**

Six academic databases were searched to identify relevant peer-reviewed journal articles published in English between July 2011 and June 2022. Studies were included if they reported on healthcare program sustainability and explicitly identified barriers to, and facilitators of, sustainability.

**Study design:**

A systematic integrative review guided by the Preferred Reporting Items for Systematic Reviews and Meta-Analysis (PRISMA) statement. Study quality was appraised using Hawker’s Quality Assessment Tool.

**Data collection/extraction methods:**

A team of reviewers screened eligible studies against the inclusion criteria and extracted the data independently using a purpose-designed Excel spreadsheet. Barriers and facilitators were extracted and mapped to the Integrated Sustainability Framework (ISF). Frequency counts of reported barriers/facilitators were performed across the included studies.

**Results:**

Of the 124 studies included in this review, almost half utilised qualitative designs (*n* = 52; 41.9%) and roughly one third were conducted in the USA (*n* = 43; 34.7%). Few studies (*n* = 29; 23.4%) reported on program sustainability beyond 5 years of program implementation and only 16 of them (55.2%) defined sustainability. Factors related to the ISF categories of inner setting (*n* = 99; 79.8%), process (*n* = 99; 79.8%) and intervention characteristics (*n* = 72; 58.1%) were most frequently reported. Leadership/support (*n* = 61; 49.2%), training/support/supervision (*n* = 54; 43.5%) and staffing/turnover (*n* = 50; 40.3%) were commonly identified barriers or facilitators of sustainability across included studies. Forty-six (37.1%) studies reported on the outer setting category: funding (*n* = 26; 56.5%), external leadership by stakeholders (*n* = 16; 34.8%), and socio-political context (*n* = 14; 30.4%). Eight studies (6.5%) reported on discontinued programs, with factors including funding and resourcing, poor fit, limited planning, and intervention complexity contributing to discontinuation.

**Conclusions:**

This review highlights the importance of taking into consideration the inner setting, processes, intervention characteristics and outer setting factors when sustaining healthcare programs, and the need for long-term program evaluations. There is a need to apply consistent definitions and implementation frameworks across studies to strengthen evidence in this area.

**Trial registration:**

https://bmjopen.bmj.com/content/7/11/e018568.

**Supplementary Information:**

The online version contains supplementary material available at 10.1186/s13012-023-01315-x.

Contributions to the literature

- Despite a growing number of studies on sustainable healthcare, previous reviews typically do not report barriers to and facilitators of the sustainability of healthcare programs.
- Previous literature lacks consistent definitions of sustainability and working definitions of factors associated with sustainability, limiting the ability to accurately assess the sustainability of implemented programs.
- Building on the Integrated Sustainability Framework, this review provides new working definitions applied in assessing barriers and facilitators to maintaining healthcare programs.
- This is the first review of sustainability to assess discontinued healthcare programs and identifies factors leading to discontinuation.

## Background

Healthcare system sustainability is the ongoing capacity to deliver affordable and effective care that contributes to better health outcomes over time. There are many threats and challenges to the sustainability of healthcare systems across the world, including an ageing population, increasing costs of delivering healthcare, costly new medical technologies and growing consumer demand [1–4]. Crises, including natural disasters, large-scale accidents, epidemics, and pandemics add further challenges to already over-stretched healthcare systems [5, 6]. A sustainable healthcare system is one that is also resilient, such that it constantly adapts and endures despite these ever-changing pressures while maintaining performance in terms of health outcomes [7].

As healthcare systems strive towards delivering value-based care within these challenging contexts, improvement programs to increase quality, safety, effectiveness, and efficiency of healthcare have proliferated [8]. Quality improvement programs are now ubiquitous across healthcare sectors and facilities. Such programs are important to support innovations where new and more effective health technologies, models of care delivery, and financing are adopted and ideally, while old ineffective and inefficient ones are phased out.

To sustain the benefits from innovations in healthcare, innovations must be empirically evaluated to ensure they are indeed effective and deliver the outcomes that they promise, at scale and across different contexts. However, large-scale innovations are rare in healthcare systems and most innovations consist of improvement projects that tend to be short-term and proscribed—implemented in single centres or regions [1, 2]. Despite its importance, the sustainability of implemented improvement programs is under-researched with a limited evidence base to support decisions [9]. For example, discontinuing effective programs because of a lack of ongoing investment is wasteful and unethical [10]. Our understanding of how and why programs implemented in the real-world are sustained or discontinued is also limited, often because evaluations of improvement programs are almost always performed over the short term [11, 12]. The need for continuing investment in effective programs is well recognised as an important factor of sustainability, however, it is not the only factor [11, 12]. Shelton et al. [12] proposed the Integrated Sustainability Framework (ISF), which identifies important factors that help or hinder program sustainability. The ISF includes inner contextual factors (i.e., program champions, leadership/support, organisational resources/funding, staffing/turnover) and outer contextual factors (i.e., socio-political context, funding environment, external leadership, and values, needs and priorities), characteristics of the interventions or programs (i.e., perceived benefit/need, adaptability, and fit with context and population), characteristics of people or institutions implementing these programs (i.e., implementer/provider characteristics, implementer skills/expertise) and the processes used for implementation (i.e., partnership/engagement, training/supervision, program evaluation/data, adaptation) [12].

Healthcare system sustainability as applied to programs that are implemented in the healthcare delivery system are poorly defined and understood conceptually. In their systematic review of 125 studies of program sustainability published up until 2011, Stirman et al. [11] identified gaps in the application of definitions of sustainability when developing and implementing programs. Studies included in their review seldom reported definitions in sufficient detail to be able to assess sustainability in a systematic manner [11]. A recent systematic integrative review [1] and scoping review [2] also found gaps and inconsistencies around the definitions of program sustainability, with less than 30% of studies providing the definitions in both reviews. Sustainability was often discussed as an extension of implementation, with many studies reporting that at the end of a 1-to-2-year implementation project, the program “was sustainable” without providing long-term outcomes or specific measures of sustainability [1].

### Rationale

The factors that act as barriers/facilitators for the sustainability of healthcare programs are inadequately reported and are poorly understood. The determinants of successful implementation are often reported; however, these are likely to be quite different to the factors related to sustainability [10, 13]. For example, factors such as trialability, intervention fidelity and factors associated with the inner setting are often talked about with reference to successful implementation. Different factors are more likely to be important for health program sustainability and program scaling, including outer setting factors such as socio-political and funding environment, external leadership, and values, needs and priorities of communities and populations, data and evaluation to demonstrate value and to support adaptations as contexts change [13]. Stirman and colleagues [11] identified gaps in research on public health program adaptations and factors that drive sustainability such as organisational context and capacity, processes and characteristics of implemented programs. A deeper understanding of barriers to, and facilitators of, program sustainability is essential to support the development, implementation, and evaluation of innovative healthcare programs, to support decision-making around program continuation, adaptation, scale-up, and diffusion, and to maximise the long-term benefits of programs. It is similarly important to understand the encountered barriers and contexts that lead to program discontinuation. To develop this understanding, a review and synthesis of current knowledge, guided by a theoretical framework such as the ISF [12] is needed.

### Objectives

Our primary objective was to build on the studies of Stirman et al. [11] and Braithwaite et al. [1] to identify the barriers and facilitators associated with the sustainability of implemented and evaluated improvement programs in healthcare delivery systems, and then to map them to the ISF. Our study also aimed to discern the extent to which the discontinuation of healthcare programs was reported in the literature and to identify factors that led to these programs being discontinued.

## Methods

The review forms part of a body of research investigating the sustainability of healthcare programs, seeking to bring it up to date [1]. The search strategy, study selection and quality assessment mirror those outlined in a published integrative review on this topic [1], with the present updated review conducted in June 2022. The data relating to barriers and facilitators, the analysis and synthesis of data, the results, and the implications and conclusions drawn from these findings are unique to the current review. The review was guided by the Preferred Reporting Items for Systematic Review and Meta-Analyses (PRISMA) statement (Table S1) [14].

### Protocol and registration

The published protocol for this review can be found at https://bmjopen.bmj.com/content/7/11/e018568 [15]. Modifications to the protocol have been previously published and the details of the updated search are described here [1].

### Search strategy

The search strategy was developed in consultation with two medical librarians and included six academic databases: CINAHL, EMBASE, Ovid MEDLINE, Emerald Management, Scopus and Web of Science [1]. Additional studies were identified by hand searching reference lists of relevant systematic reviews. The search strategies for all databases are provided in Table S2*.*

### Study selection

The selection process has been previously described [1]. The reviewers had varying degrees of experience in conducting systematic reviews, and 13 out of the 16 authors had previously been an author on at least one systematic review study. A blinded review of 5% of titles and abstracts was undertaken, and discrepancies were discussed among the reviewers (KL, LT, HA, JHD, GL, EM, KH, AC, CLS, LVB, LAE, and GD), with two reviewers (YZ and JB) acting as arbitrators, until a consensus was reached. The remaining screening of abstracts and titles was undertaken in Rayyan [16], a web and mobile app for systematic reviews, according to the inclusion criteria with records randomly allocated among the reviewers [1]. Publications were assessed against the following inclusion criteria: (1) English-language, (2) peer-reviewed journal article, (3) primary empirical research, (4) published July 2011–June 2022, (5) healthcare setting, (6) evaluation of program, (7) assessment of program sustainability, and (8) focus on changes/improvements to the healthcare system.

Studies reporting on public health programs including population-based prevention programs, community-level outcomes only, or patient-level outcomes only were excluded. Studies that did not identify barriers to, or facilitators of, program sustainability, and studies that reported barriers or facilitators of implementation only were also excluded. Studies included at the abstract-review stage were re-assessed against the inclusion criteria during full-text review. Based on our previous integrative review [1] of 92 studies and drawing on the work of Stirman et al. [11], Shelton et al. [12], and Scheirer and Dearing [10], health program sustainability was conceptualised from a systems or organisational view-point. Therefore, studies were included if they reported on the following:

- Evaluation of a program after funding had ended, or after the initial staff training or implementation phase; and
- Explicitly assessed sustainability, either using qualitative, quantitative, or mixed methods, for example, stakeholders’ views of sustainability, evidence of ongoing care delivery under the program, ongoing funding; or,
- Longitudinal studies, for example, evaluations conducted over multiple time points.

### Data collection processes and data items

Following previously-described work [1], a purpose-designed Excel spreadsheet was utilised for data extraction. The spreadsheet was piloted by reviewers on two studies. The remaining studies were randomly assigned to the reviewers for data extraction. Verification of the accuracy and meanings of the extracted data was undertaken independently by seven reviewers (LT, AC, PNAD, CLS, NH, and YZ). Any discrepancies were resolved through team discussions during regular meetings (over 10 group meetings were held).

### Data analysis and synthesis

It was often not possible to classify factors influencing sustainability in a binary way, i.e., as either a barrier or facilitator. For example, the degree to which a program was sustained may have been influenced by a high (facilitator) or low (barrier) level of leadership. As such, barriers and facilitators were conceptualised as part of a single construct, representing two ends of a spectrum.

Barriers/facilitators of program sustainability were synthesised using the ISF [12]. The ISF embodies 36 “emerging factors” grouped together under five categories: outer setting, inner setting, intervention characteristics, processes, and implementer and population characteristics. Shelton et al. [12] did not provide definitions for each of their emerging factors making it difficult for the reviewers to classify some of the barriers/facilitators. To overcome this challenge, working definitions for the ISF emerging factors were developed by two reviewers (LT and HA) based on relevant literature and other frameworks including Weiner et al. [17] and the Consolidated Framework for Implementation Research (CFIR) [18]. The proposed definitions were discussed with the broader review team before being applied during data analysis and interpretation (Table 1). Frequency counts were then performed for each emerging factor (Table 2), in addition to a qualitative narrative synthesis. For the purpose of this review, emerging factors will henceforth be referred to as “barriers/facilitators”.

**Table 1:** Definitions/operationalisations of emerging factors in the Integrated Sustainability Framework

Constructs and emerging factors	Definitions/operationalisations	Source
**Outer setting**	The external contextual factors that may influence the sustainability of interventions	Adapted from Shelton et al. [12]
Policy and legislation	External policy and legislation (governmental or other central entity) to spread interventions	Adapted from CFIR [18] (External Policies and Incentives)
Socio-political context	The influence of the local context in which the intervention is delivered	Adapted from Shelton et al. [12]
Funding environment	The availability and stability of additional external or on-going funding necessary to deliver an intervention beyond the implementation period	Definition developed by authors
Leadership	The influence of external leadership (e.g., government, senior manager/executives of health services or hospitals) to the setting in which the intervention is delivered	Definition developed by authors
Values, priorities, needs	The degree of fit between intervention activities and the values, priorities, and needs of stakeholders (e.g., policymakers, health departments, communities/society and populations)	Definition developed by authors
Community ownership	Levels of community support and trust in the intervention	Adapted from Shelton et al. [12]
**Inner setting**	Organisational factors that may influence the sustainability of interventions	Adapted from Shelton et al. [12]
Funding/resources	The availability of resources dedicated to intervention delivery, e.g., funding for staff, equipment, consumables, staff training	Adapted from CFIR [18] (Available resources)
Leadership/support	Active participation in and accountability to intervention delivery by leaders and managers	Adapted from CFIR [18] (Leadership engagement)
Climate/culture	Climate: “The absorptive capacity for change, shared receptivity of involved individuals to an intervention, and the extent to which use of that intervention will be rewarded, supported, and expected within their organisation.” Culture: “Norms, values, and basic assumptions of a given organisation”	Direct quotation from CFIR [18] (Implementation climate and Culture)
Staffing/turnover	The degree of stability of the organisation’s workforce as it relates to the delivery of the intervention	Definition developed by authors
Structural characteristics	The social and functional characteristics of an organisation	Adapted from CFIR [18]
Capacity	The organisational availability of resources necessary to deliver an intervention (additional to cost of the intervention – see below)	Definition developed by authors
Champion	An individual who commits themselves to steering the implementation of an intervention and overcoming organisational resistance	Adapted from CFIR [18]
Policy (alignment)	The degree of fit between intervention activities and internal organisational policy	Definition developed by authors
**Intervention Characteristics**	The key attributes of interventions that may influence the sustainability of interventions	Adapted from CFIR [18]
Adaptability	“The degree to which an intervention can be adapted, tailored, refined, or reinvented to meet local needs”	Direct quotation from CFIR [18]
Fit with population and context	“The degree of tangible fit between meaning and values attached to the intervention by involved individuals, how those align with individuals’ own norms, values, and perceived risks and needs, and how the intervention fits with existing workflows and systems”	Direct quotation from CFIR [18] (Compatibility)
Benefits/need	“Perceived benefit/need” of the intervention	Adapted from Shelton et al. [12]
Burden/complexity	“Perceived difficulty of implementation, reflected by duration, scope, radicalness, disruptiveness, centrality, and intricacy, and number of steps required to implement”	Direct quotation from CFIR [18]
Trialability	“The ability to test the intervention on a small scale in the organisation, and to be able to reverse course (undo implementation) if warranted”	Direct quotation from CFIR [18]
Cost	“Costs of the intervention and costs associated with implementing and sustaining the intervention including investment, supply, and opportunity costs”	Direct quotation from CFIR [18]
**Processes**	Key components of the processes that may influence the sustainability of interventions	Definition developed by authors
Partnership/engagement	The use of collaborative partnerships and stakeholder engagement to support the implementation and sustainability of an intervention	Adapted from Shelton et al. [12]
Training/support/supervision	Provision of staff and implementer training, support and supervision to facilitate implementation and sustainment	Definition developed by authors
Fidelity	“The degree to which an intervention or program is delivered as intended”	Direct quote from Carroll et al. [19]
Adaptation	“The degree to which an evidence-based intervention is changed to fit the setting or to improve fit to local conditions”	Direct quote from Shelton et al. [12]
Planning	“The degree to which a scheme or method of behaviour and tasks for implementing and sustaining an intervention are developed in advance, and the quality of those schemes or methods”	Direct quotation from CFIR [18]
Team/board functioning	The extent and quality of collaborative and functioning relationships of the teams and boards involved in implementation and sustainment of interventions	Definition developed by authors
Program evaluation/data	The use of evaluation and data to provide feedback on performance and outcomes to be used to support processes for implementation and sustainability	Definition developed by authors
Communication	The extent and quality of communication about the intervention and its implementation among involved stakeholders	Definition developed by authors
Technical assistance	Availability of technical assistance to support the implementation and sustainment of interventions	Definition developed by authors
Capacity building	“Activities that build durable resources and enable the recipient community to continue the delivery of an evidence-based intervention”	Direct quotation from Shelton et al. [12]
**Implementer and population characteristics**	Attributes of implementers and population that may influence the sustainability of interventions	Definition developed by authors
Provider/implementer characteristics	Attributes of the provider/implementer of the intervention	Definition developed by authors
Implementation skills/expertise	The implementation skills and expertise of the individuals involved in the implementation	Definition developed by authors
Implementer attitudes	General attitudes of the implementing group towards the intervention	Definition developed by authors
Implementer motivation	The degree to which implementers are motivated (willing) to implement and sustain the intervention. This construct relates to organisational readiness for change which refer to organisational members’ motivation and capability (i.e., being willing and able) to implement intentional organisational change	Adapted from Weiner et al. [17]
Population characteristics	Attributes of the population which the intervention targets	Definition developed by authors

**Table 2:** Number of included papers reporting on barriers to and/or facilitators of program sustainability^a^, organised according to the Integrated Sustainability Framework [12]

Outer setting (*n* = 46)	Inner setting (*n* = 99)	Intervention characteristics (*n* = 72)	Processes (*n* = 99)	Implementer and population characteristics (*n* = 44)
Funding environment (*n* = 26)	Leadership/support (*n* = 61)	Fit with population and context (*n* = 37)	Training/support/supervision (*n* = 54)	Implementer attitudes (*n* = 23)
Leadership (*n* = 16)	Staffing/turnover (*n* = 50)	Adaptability (*n* = 29)	Communication (*n* = 40)	Implementation skills/expertise (*n* = 21)
Socio-political context (*n* = 14)	Climate/culture (*n* = 42)	Benefits/need (*n* = 26)	Program evaluation/data (*n* = 40)	Provider/implementer characteristics (*n* = 18)
Values, priorities, needs (*n* = 11)	Funding/resources (*n* = 37)	Burden/complexity (*n* = 19)	Partnership/engagement (*n* = 35)	Implementer motivation (*n* = 12)
Policy and legislation (*n* = 11)	Champion (*n* = 31)	Cost (*n* = 12)	Adaptation (*n* = 24)	Population characteristics (*n* = 8)
Community ownership (*n* = 9)	Capacity (*n* = 30)	Trialability (*n* = 3)	Team/board functioning (*n* = 23)	
	Structural characteristics (*n* = 19)		Planning (*n* = 21)	
	Policies (alignment) (*n* = 13)		Capacity building (*n* = 21)	
			Technical assistance (*n* = 12)	
			Fidelity (*n* = 8)	

^a^Details of individual studies categorised by ISF category and all factors under each category are provided in Table S4

To identify critical barriers to program sustainability and how these might be overcome, one part of our analysis focused on programs that were discontinued. To identify critical facilitators of longer-term sustainability, the review also focussed on reports of programs that were sustained for at least 5 years after funding, training, or the implementation period ended, rather than the more commonly reported time points of 1 to 3 years at the end of trial funding when it is difficult to separate factors related with implementation from those related with sustainability [1]. There are currently no specific agreed or pre-determined time points at which a program is deemed to be sustainable. Thus, informed by the literature, especially published reviews [1, 11, 12, 20, 21] and, after team discussions, we concentrated on programs that had been sustained for 5 years or longer after funding, staff training or the implementation period or the trial had ended.

### Quality assessment

The quality of included studies was assessed by ten reviewers (KL, LT, HA, JHD, GL, AC, PNAD, CLS, GD, and NS) using Hawker’s Quality Assessment tool [22] and Lorenc et al.’s quality ratings [23] (low, medium, high). A blinded quality assessment of a randomly selected 6% (*n* = 7) sample of included studies was conducted to ensure consistency of ratings among the reviewers. The remaining studies were randomly assigned to individual reviewers and any queries were discussed and resolved in a team meeting.

## Results

### Study selection

**Fig. 1:**
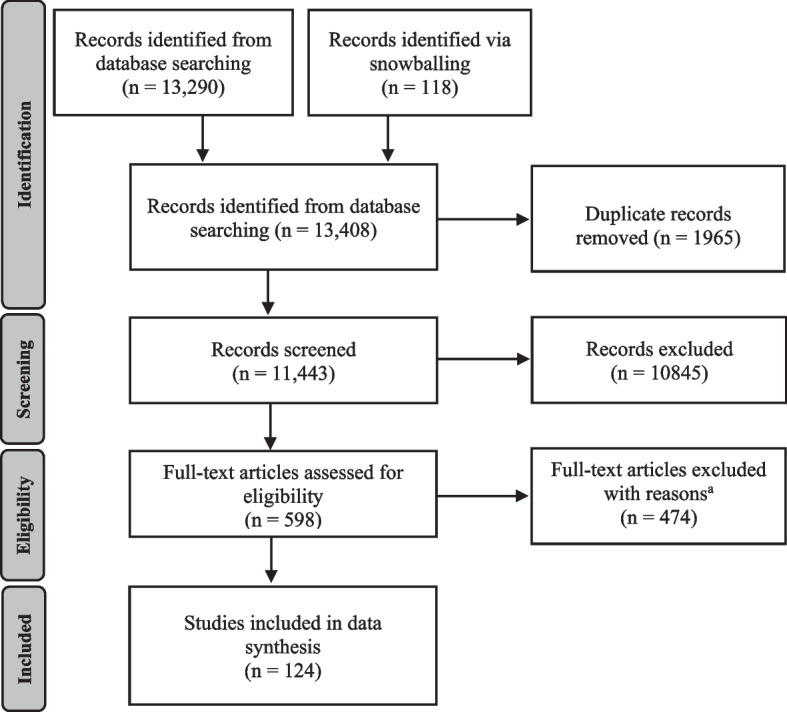
PRISMA flowchart of studies identified for relevant studies for inclusion in the review

A total of 11,443 studies were screened after duplicates were removed. At the title/abstract review stage, 10,845 out of the 11,443 were excluded, leaving 598 studies progressing to full-text review, with 124 studies being retained for data analysis and synthesis (Fig. 1). The main reasons for exclusion at full-text review were that the publication did not assess sustainability or discontinuation of a program (*n* = 185), did not focus on change improvements in the healthcare system (*n* = 91), or no evaluation of a program was reported (*n* = 102).

### Study characteristics and quality assessment

Fifty-two studies (41.9%) reported qualitative results, 37 (29.8%) used mixed methods and 35 (28.2%) reported quantitative results. Most studies were longitudinal with assessment of outcomes at different time points (*n* = 55; 44.4%), in addition to case studies (*n* = 32; 25.8%), and cross-sectional studies (*n* = 30; 24.2%). Thirty-five countries were covered by the 124 studies, with seven including more than one country. The majority of studies originated from the USA (*n* = 43; 34.7%), Canada (*n* = 14; 11.3%), the United Kingdom (*n* = 11; 8.9%), and Australia (*n* = 11; 8.9%). Eighty-four studies scored 30–36 points (high quality), 35 scored 24–29 points (moderate quality), and 5 scored less than 24 points and were considered low quality on Hawker’s Quality Assessment Tool [22, 23] (Table S3). No studies were excluded as all studies were deemed as providing sufficient information related to sustainability.

### Barriers and facilitators

Table 2 summarises the number of studies that explicitly referred to the barriers/facilitators under the ISF as defined in Table 1. Detailed data for each barrier/facilitator including frequency counts are provided in Table S4. The most commonly identified barriers/facilitators were related to the ISF inner setting category (*n* = 99; 79.8%) and the processes category (*n* = 99; 79.8%), (Table 2).

#### Inner setting

Barriers/facilitators related to the inner setting were reported by 99 (79.8%) studies (Table 2). Organisational factors commonly identified by studies as influencing intervention sustainability were leadership (*n* = 61; 61.6%), staffing/turnover (*n* = 50; 50.5%), climate/culture (*n* = 42; 42.4%), and funding (*n* = 37; 37.4%). Committed leadership, especially from formally appointed leaders, was reported to play a critical role in sustaining interventions [24, 25]. Brewster et al. [26] described the presence of a small number of key staff members to maintain an intervention in place and demonstrable commitment by management over time as important facilitators. Staff turnover was often cited as a barrier to continuation of interventions, which related to the need to re-train new staff whilst dealing with staff shortages to deliver the program [25, 27–29].

The climate/culture of the inner setting were also commonly discussed, as these factors created a supportive environment for change implementation. Supportive work culture [30] and providing rewards and recognition [27, 31] were recognised facilitators. Conversely, factors including lack of a unified identity and poor accountability [32], and staff and institutional resistance [33, 34] acted as barriers to sustainability. Availability of funding facilitated the delivery of an intervention, independent of the cost of the intervention itself, however, inadequate resources to support and expand interventions presented a barrier to continuation [35].

#### Processes

The processes related to program sustainability were reported by 99 (79.8%) studies and included the availability of training/supervision (*n* = 54; 54.5%), program evaluations and data (*n* = 40; 40.4%), and communication (*n* = 40; 40.4%) (Table 2). The provision of regular staff training about new programs, for both newly hired staff and experienced staff members, was an identified facilitator for program sustainability [25, 35–37] whereas a lack of training was identified as a barrier [25, 35, 38]. The importance of program evaluation and regular feedback of data concerning program outcomes to staff members involved in the implementation and to stakeholders, was highlighted by at least seven studies [34, 39–44]. Positive program outcomes that were regularly communicated and visible to staff members were linked with program sustainability [37, 45–47] and the converse hindered program sustainability [48, 49].

Efficient and ongoing communication among stakeholders involved in the program [24, 31, 50] and strong collaborative partnerships facilitated program sustainability [51–53]. Clear roles and responsibilities as well as mutual trust among stakeholders to fulfil their unique responsibilities were also reported as facilitators [50, 54]. Decreased communication among stakeholders after program implementation was a recognised barrier, for example, limited communication after implementation of a multidisciplinary hospital‐based surgical program threatened sustainability in some locations [55].

#### Intervention characteristics

Intervention characteristics were reported in 72 studies (58.1%), with a good “fit” and alignment of interventions with existing systems and local contexts facilitating sustainability reported in 37 studies (51.4%). For example, facility-based consultations in eye care interventions in Ghana were more likely to be routinised due to a high level of compatibility with the hospitals’ mandate, whereas outreach activities were less likely to be sustained due to a low level of compatibility and lack of role clarity [56]. Intervention adaptations to overcome challenges in resource-limited settings bolstered sustainability by improving fit with the population’s needs and context. For example, across Uganda, shifting intervention delivery from physicians to other staff (nurses and pharmacists) in an anti-retroviral therapy program and adopting greater task sharing with non-physician staff, supported program sustainability [57]. Urquhart et al. [58] also reported the importance of adapting interventions to improve fit with cancer survivors’ needs in different settings, including transitioning to online delivery and tailoring of tools.

Furthermore complex interventions were less sustainable [24, 59], while simpler interventions were reported to be more sustainable [26, 53, 60].

#### Outer setting

Factors related to the outer setting category were reported by 46 (37.1%) studies, with funding (*n* = 26; 56.5%), external leadership by stakeholders (*n* = 16; 34.8%) and socio-political context (*n* = 14; 30.4%) reported most frequently (Table 2). Support and leadership from external stakeholders facilitated intervention sustainability [35, 61, 62]. External contextual factors, such as funding environment and socio-political context, were found to both positively and negatively influence intervention sustainability. For example, Bond et al. [35] found that adequate financing facilitated sustainability, whereas Fleizer et al. [32] and Olumide et al. [29] reported that insecure sources of funding challenged the continuation of programs. A high dependence on time-limited funding from external donors created a barrier to sustainability, especially when other funding sources were not planned for in the longer term [62, 63].

Ongoing involvement of leadership in the region [49] and at national level [61] were thought to facilitate sustainability while a lack of support from governments created barriers to program sustainability [38, 64]. Furthermore, the socio-political context was reported as an influencing factor by 14 studies. For example, De Neve et al. [64] found that political turnovers and instability led to discontinuation of a program as ‘political actors’ and priorities changed. A mismatch between the program activities and values, priorities and needs were also identified as a barrier [64–66]. Socio-political factors were also reported as important facilitators. For example, political and financial stability and perceived value of the implemented programs among external stakeholders were thought to support the sustainability of HIV/AIDS relief programs [67]. In addition, joint planning between the donor, non-governmental organisations, health facilities and government enabled stakeholders, especially local governments, develop a better understand their health system needs and therefore to sustain effective health investments [67]

#### Implementer and population characteristics

Factors related to implementer and population characteristics were reported by 44 studies (35.5%), and half of them reported that general attitudes of implementers of new programs (*n* = 23; 52.3%) both positively and negatively affected sustainability (Table 2). Implementers being realistic in their expectations, including realistic timelines, adequate resourcing, ongoing engagement with staff delivering the program, and with program recipients, facilitated sustainability [65]. Staff members’ beliefs about the advantages of programs facilitated sustainability [47, 55, 68], whereas negative attitudes and fear of change were barriers [37, 69, 70]. Staff members who perceived that a new program would have negative consequences for their autonomy and workload was identified as a barrier [27, 69].

The right skills and level of expertise were also identified as implementer characteristics that positively influenced the delivery of sustained programs. For example, a rural volunteer program in Canada underlined that volunteer coordinators with sufficient skills and expertise, who also trained and mentored others, was an important facilitator [36]. However, Fox et al. [48] found that underuse of highly experienced and skilled staff might lead to job dissatisfaction and staff attrition, posing a barrier. For example, emergency nurses were concerned about deskilling and underutilisation after acquiring new high-level skills which were not required to care for low acuity patients [48].

### Identifying barriers that resulted in program discontinuation, and facilitators of long-term program sustainability

Eight of the 124 studies (6.5%) explicitly referred to the discontinuation of programs [24, 29, 35, 71–75]. Table 3 summarises factors associated with discontinuation, including lack of financial viability; workforce issues (lack of trained workforce, strict role boundaries, competing demands on staff time, poor preparation, training and planning); and lack of engagement and misunderstandings between implementers and staff expected to deliver the program. Misalignment with existing policies and workflows, lack of ongoing support from the implementation team and multiple changes being implemented at the same time also contributed to program discontinuation (Table 3).

**Table 3:** Summary of studies reporting on programs that were discontinued, illustrating common barriers of sustainability (*n* = 8)

Study details	Setting	Program	Evaluation timeframe	Details of discontinuation	Barriers to sustainability
Bond et al. [35]	49 community mental health sites (USA)	The National Implementing Evidence-Based Practices Project	2 years and 6 years	Eighteen sites (37%) discontinued the project, seven of which were discontinued ≤ 2 years. An additional eight sites (16%) discontinued the project then reinstated it after a lapse of time	• Financial barriers, e.g., state and national budgetary policies, agency finances/resources, and general economic trends • Lack of support (prioritisation) from staff for the evidence-based practices • Lack of training or appropriately credentialed practitioners and supervisory staff • Workflow barriers such as documentation burden, physical environment, nonfinancial policies
Lean et al. [71]	Sample of ten inpatient mental health rehabilitation units, representing 50% of units that had received the intervention (UK)	Rehabilitation Effectiveness for Activities for Life (GetREAL): Staff training intervention to improve patient engagement in activities	1 year	Despite initial improvements in several workforce outcomes, the skills that staff members had gained and the agreed implemented processes and structures were not sustained after the implementation team left	• Insufficient preparation for the intervention in which there was a lack of information prior to the arrival of the training team • Engagement with the intervention team was seen by staff to be too short to achieve its intervention goals • Some staff members misunderstood the aims of the intervention, thinking the training was aimed at patients • Lack of resources and time • Staff members considered intervention activities to be outside their role responsibilities once the intervention team left • Champions were signed up by management as opposed to volunteering, people were unaware who champions were, and the champion role was seen to carry additional burden with no rewards • The prioritisation of mandatory paperwork competed with patient engagement
Olumide et al. [29]	10 of the NURHI-1 intervention facilities in Ilorin and six in Kaduna (Nigeria)	The Nigerian Urban Reproductive Health Initiative	3 years	The programme ended in Ilorin	• Frequently stock-out of family planning commodities and consumables • Lack of funding to finance the program • Lack of advertisements • Non-existent of NURHI-type training (discontinued) • Lack of trained staff due to turnover, retired, relocation
Peterson et al. [72]	49 sites that implemented Evidence-Based Practices (EBPs) (USA)	The National Implementing Evidence-Based Practices Project	2, 4, 8 years	Year 2: 10 sites discontinued Year 4: 16 sites discontinued Year 8: 8 sites discontinued	• EBPs implemented with high fidelity at sites engaged in agency-level strategies to support sustainability remained vulnerable to discontinuation • Leadership/supervisor turnover • External funding and supportive entities affect long-term survival
Pomey et al. [73]	Five Canadian health care organisations (Canada)	The Wait Time Management Strategies (WTMS) implementation	18 months	One of five cases was deemed non-sustainable because the WTMS failed to reduce waiting times to less than 26 weeks within 18 months (Case 1: Atlantic Canada)	• Lack of incentives to encourage staff engagement • Cultural gap between senior and junior surgeons • Lack of engagement among physicians • Lack of standardisation of the referral process resulting in delays • Nursing shortages due to budget constraints • Physicians competing with other specialties for the operating rooms • Lack of funding
Seppey et al. [74]	Six community health centres and two referral health centres (Mali)	A pilot project to improve demand and supply of health services through financing performance in targeted services	Unclear	The project was deemed to have a weak level of sustainability with many of the project activities being discontinued or diminished at the end of the project	• Insufficient and unstable resources (financial, human, material) • A lack of supervision • Distinction between the different roles (management vs. healthcare provision) led to a loss of contact between stakeholders • Planning focused on potential scaling up and outcomes rather on the sustainability of the project/routines • High rotation of personnel • Lack of involvement of local authorities • Insufficient time to integrate routines
Vidgen et al. [24]	16 study sites (eight per state) implementing PEACHQLD in Queensland and Go4Fun in NSW (Australia)	Childhood obesity management services within two Australian States (New South Wales and Queensland)	Queensland Department of Health ceased funding and coordinating in 2017. Data collection timeline was unclear	Three out of eight sites had discontinued implementation in NSW whereas one of five sites had discontinued in Queensland. Eventually, all local service providers decided to discontinue the program in 2017	Perceived negative influence: Within-State: Queensland • Complexity of implementing the program (the strongest factor) • Acknowledgement of the need (tension) for change • Alignment with external policy and incentives • Program champions within the organisation • Evaluation and feedback processes • Leadership engagement Discontinued sites in Queensland • The high costs of delivering and participating in service in remote areas • Compatibility and not meeting patient needs • Geographic location Discontinued sites in NSW • Complexity, cost, patient needs and resources • Identification by program delivery staff with the organisation
Zakumumpa et al. [75]	Six health facilities receiving donor support for implementing the ART program (Uganda)	Uganda National antiretroviral therapy (ART) scale-up program	6 years	Two (of six) sites selected for analysis had high sustainability, two had low sustainability, and two were ‘non-sustainers’	• Scale-up was at odds with organisational for-profit orientated goals • Higher patient loads led to greater indirect costs, long waiting times and high workloads • Staffing shortages were coupled with increased patient demand due to the introduction of donor-supported free ART services • Irregular drug supplies • High ART-proficient staff turnover • Lack of staff motivation due to low salaries and dissatisfaction with rewards • Remoteness of location associated with local infrastructure barriers, e.g., poor road network linkages and electricity supply

A total of 29 studies (23.4%) reported that the programs had been sustained for 5 years or longer (Table S5). Five example studies reporting on programs that were sustained for 5 years or more are summarised in Table 4. These five example programs were selected because they demonstrate a wide variety of factors that supported program sustainability. Eight of the 29 studies (27.6%) reported that program adaptability and/or adaptation were the key facilitators of long-term program sustainability. For example, most health facilities implementing a multi-site anti-retroviral therapy (ART) scale-up program in Uganda modified and tailored the intervention in order to improve fit with their resource-constrained conditions thereby fostering long-term sustainability between 2004 and 2014 [57]. Another study by Oliveira et al. [76] highlighted that ongoing monitoring and adaptation of the Family Health Program (FHP) in response to critical events were deemed as strategic facilitating factors for the sustainability of the program for 12 years.

**Table 4:** Five examples describing programs sustained for 5 years or more and illustrating common facilitators of sustainability^a^

Study details	Setting	Program	Evaluation timeframe	Details of sustainability	Facilitators of sustainability
Ament et al. [77]	Four hospitals, breast cancer surgery services (the Netherlands)	Short-stay program in breast cancer surgery care	5 years	The compliance with recommendations to facilitate short stay after breast cancer surgery increased from 65% after implementation to 78% 5 years post-implementation	• Normalization of short-stay breast cancer surgical protocols • The organisational culture in early adopter hospitals may have facilitated the sustained results • Adaptability of the new program and healthcare workers’ compliance with the key program recommendations
Casati and Bjugn [60]	One pathology department implementing a national initiative (Norway)	A national electronic template for histopathology reporting of colorectal carcinoma resections implemented into daily routine practice	5 years	The template was used in 91.8% of cases (*n* = 1186) and had significantly improved upon the reporting of parameters at 5 years post-implementation	• Template clarity and simplicity • Health leaders as champions • Culture of support for the template from staff members and peers • Ongoing national funding • Changes in individual long-term behaviour to refer to the national electronic template and include key parameters when writing reports
Magadzire et al. [78]	The Western Cape Department of Health (WCDoH) (South Africa)	Chronic Dispensing Unit (CDU)	10 years	Started with eight centres in 2005 and reached 216 centres by 2015	• Leadership and support from funders • Engagement with clinical staff involved in developing the program • Partnership between the WCDoH and the contractor (UTI Pharma) • Adaptability and flexibility between involved stakeholders, e.g., adapting to policy changes • Policy alignment at multiple organisational levels, and with the implementation team (health personnel and the contractor) • Technological advancements (e.g., machines allowing the picking, packaging and labeling of medicines) that increased scope of program • Funding for 5 years from government and contractors through political alignment
Oliveira et al. [76]	Primary care—over 39,000 family health units [FHUs]) (Brazil)	The Family Health Program (FHP) implemented in 1994 as national policy	12 years	Six critical events that allowed the program to evolve and change in response to its context, thus facilitating long-term sustainability	• The intervention was compatible with the government’s vision, allowing it to withstand policy and political changes occurring in local health management • The change of municipal management by mobilising champions to rebuild alliances with other sectors for collaboration • The involvement of staff as decision makers and negotiators to maintain the stability of the intervention • Ongoing monitoring and adaptation of the program in response to critical events that occurred in the 11 years since the program’s inception, e.g., staff turnover and navigating different council approaches to healthcare
Zakumumpa et al. [25]	Four health facilities were purposively selected, with two sites with the highest institutionalisation scores and two the lowest institutionalisation scores. (Uganda)	Anti-retroviral therapy (ART) scale-up	Implementation history of between 8 and 11 years (sites with the highest scores)	Two sites with the highest institutionalisation had successfully transitioned to a more ‘permanent’ state in ART service delivery whereas the two sites with the lowest scores were in ‘pilot’ mode	• The availability of written procedures and manuals for ART service delivery and organisational strategic plans • Adaptations to program models when needed • Formally appointed roles • The presence of program champions • Service support from other departments • ART program was embedded in daily organisational routines • Deliberate strategies for training and retention of ART-proficient staff • Salary support for key staff in the ART clinic

^a^Details of all programs sustained for 5 years or more are provided in Table S5

Multi-site studies demonstrated the importance of understanding the local contexts and several studies reported that the programs were sustained in one context but not in another. Vidgen et al. [24] demonstrated such contextual differences by highlighting that the decision to outsource a program to an external provider under a limited time contract was a barrier to sustainability. On the other hand, Zakumumpa et al. [25, 75] aptly demonstrated both sustainability and discontinuation in different sites to show significant barriers (Table 3) and facilitators (Table 4) related to local context.

## Discussion

Our systematic integrative review demonstrated that the literature on the sustainability of innovations or improvement programs in healthcare is developing. Barriers and facilitators of healthcare program sustainability were identified and mapped to the ISF using our working definitions, with the most prevalent barriers/facilitators relating to inner setting (79.8%) and processes (79.8%). The review identified important gaps including limited long-term program evaluations. Studies often claimed program sustainability even at 1 or 2 years after implementation. Longer-term evaluations are needed to confirm such claims as few studies (*n* = 29; 23.4%) reported on program sustainability 5 years or more after implementation. Short-term evaluations were common which is not surprising given the approach often taken by health organisations and governments when implementing improvement programs in the healthcare system [79]. Short-term funding limits capacity to rigorously evaluate, adapt, sustain and scale programs over the longer-term.

Leadership and support emerged as a key influencer in program sustainability. Consistent with the findings from the systematic reviews by Cowie et al. [80] and Penno et al. [81] using Lennox’s consolidated framework [82], our review suggests that the support of leaders plays a critical role in achieving sustained programs. Enthusiasm and support of leaders, however, is not enough to effectively support and sustain healthcare programs without considerable skill, expertise, and capacity of these leaders. Ambitious leadership without sufficient managerial skills and technical experience can negatively impact sustainability of healthcare programs due to a loss of focus on the program after implementation [83].

Workforce issues, such as high staff turnover, were identified as common barriers to sustainability. This finding is consistent with other reviews [21, 80]. Our review identified that program discontinuation could be attributed to staff turnover associated with lack of adequate training and trained staff, lack of incentives and recognition, and competing priorities [29, 75]. In contrast, Shelton et al. [12] reported that the nature and influence of processes, including staff training, were barriers/facilitators less often reported to be associated with sustainability. Ninety-nine studies (79.8%) included in our review reported at least one factor under the processes category, with more than half mentioning training/support/supervision. The role of training was essential to equip staff with skills and knowledge required to deliver program interventions and to maintain fidelity [34, 62]. Ensuring adequate time and resources to train staff as required, and not only at the beginning of implementation, should be considered in planning new programs that are intended to last. New programs require new roles and new role descriptions, which should be developed, maintained, and updated to ensure role clarity, responsibility, and scope within the program and the context within which the program is being sustained.

Much of the literature about healthcare system sustainability is focussed on factors that make programs and systems last over time. This, of course, is sensible, however it provides a one-sided view. One of the unique aspects of our review is the analysis of factors that led to the discontinuation of programs. Understanding why programs cease is a critical complement to our understanding of factors that make programs sustainable. Increasingly, it is being recognised that many programs may continue despite becoming ineffective, inefficient, or no longer needed [84]. Therefore, the strident quest for sustainability of all implemented programs may be inappropriate and may in itself contribute to the wider system unsustainability, as maintaining ineffective, inefficient or defunct health programs can contribute to waste or low-value care [84, 85].

A greater emphasis in the literature on reporting on ineffective or unsustainable programs would enrich our understanding of factors associated with program sustainability and may also prevent others from wasting efforts and investment. However, there were only eight studies out of the 124 included in our review (6.5%) that reported on discontinuation. Publication bias, where negative results are less likely to be published, may be a factor skewing the literature towards successful ongoing programs [86]. This limited literature restricts our understanding of the factors that lead to discontinuation, or how the decision to discontinue was influenced, made, or planned. All eight studies describing discontinued programs, or parts of programs, argued that their programs should have been sustained; however, scaling-up healthcare programs must be accompanied by appropriate long-term monitoring and ongoing evaluation to ensure that decisions to sustain, adapt or discontinue are evidence-based [84].

In line with our review, two other systematic reviews applied the ISF to assess barriers/facilitators related to program sustainability [20, 21]. One of these, by Shoesmith et al. [21] provided specific factor definitions as applied in the context of schools and/or childcare services. In line with the results of Shoesmith et al. [21] and Braithwaite et al. [1] our review also found that factors predominantly related to the inner setting were reported to facilitate intervention sustainment, including leadership support. Another review by Hall et al. [20] applied the factor definitions developed by Shoesmith et al. [21] in a review of clinical, public health, and community health services. Hall et al. [20] focused on the measurement of sustainability and determinants of sustainability, and found that 28 individual measures were used among 223 articles, but only 2 of these measures specifically assessed sustainability as an outcome and one assessed both sustainability outcomes and determinants. This is an important gap also illustrated in our previous review [1], where only 6 out of 92 included studies (6.5%) reported using measures of program sustainability in healthcare delivery settings. This demonstrates a need for clear and consistent definitions and measures of sustainability that are relevant and applicable to the field being studied, in our case, healthcare. Whilst undertaking this review, it became necessary to develop working definitions guided by the literature that could be applied by the review team to operationalise the ISF and ensure consistency of interpretation of emerging factors associated with program sustainability. These new definitions should be considered and improved upon as new research emerges, to improve the consistency of assessments using the ISF.

### Strengths and limitations

Our review builds on our previous work [1] and extends the work of others [2, 11, 12] by describing in detail the factors contributing to program sustainability in healthcare settings, whilst guided by a published framework, the ISF. This review enhances evidence about the sustainability of healthcare programs by identifying and mapping barriers to and/or facilitators of a sustainability framework that support sustainability or contribute to program discontinuation. The analysis of factors that led to the discontinuation of programs is also a strength and provides important learnings to guide future healthcare program planning to avoid known barriers. Few reviews have specifically addressed program discontinuation, possibly because there are fewer publications about discontinued programs, potentially due to the known publication bias to publish positive results [86].

The application of the ISF as an underpinning theory to map barriers and facilitators of sustainability is another strength of our review, which builds on this framework by providing a working definition of each emerging factor. Importantly, we conceptualised the factors on a continuum rather than binary facilitators or barriers, for example policy and legislation may be a barrier in some settings and a facilitator in others and this may change over time. Although working definitions of emerging factors were developed for this review, they have not been applied by others to determine their validity, consistency, and applicability to the study of sustainability of healthcare programs. Further work is needed to ensure the longer-term usability or adaptation of these definitions.

Double-blinded abstract and full-text reviews were conducted in addition to many team meetings to ensure consistency of study screening, inclusion and interpretation. Interpretation of how studies conceptualised sustainability required significant discussion by the whole team especially in studies that reported on sustainability at 12 months or less after implementation—where implementation and sustainability may have been conflated. Therefore, the decision to concentrate on programs that were sustained for 5 years or more adds further validity to our findings.

The large variety of definitions of sustainability, and the failure to provide definitions of sustainability for studies that report on sustainability, has been continuously highlighted as a major limitation in previous reviews on sustainability [12, 21, 80], including in our previous reviews [1, 2]. Reviewing the literature on healthcare program sustainability was made more challenging by the large body of literature on environmental sustainability, limiting the pace of screening for inclusion and exclusion. Moreover, the heterogeneous nature of the current included studies, including lack of clear definitions or inconsistent definitions, made synthesis of the literature challenging. The exclusion of grey literature, studies published in languages other than English may mean that other relevant studies could have been missed, limiting the comprehensiveness of the evidence synthesis.

### Implication for practice and research

Our findings add to the understanding of which factors hampered or facilitated the sustainment of healthcare programs and complements previous reviews on program sustainability. The sustainability of healthcare system improvements in our review mapped to the inner setting and processes category of the ISF with leadership/support, training/support/supervision, and staffing/turnover being the most frequently reported barriers/facilitators. In line with other reviews [80, 81], the results suggest that these barriers/facilitators should be prioritised in the sustainability phase of programs and considered in light of organisational readiness and ongoing resources for program delivery. However, it should be noted that this suggestion is based on the findings from the 124 studies in our review, which were mainly located in high-income countries.

Aligned with the literature on program implementation, factors related to the inner setting and processes were commonly reported in studies included in our review. However, the outer contextual factors such as the socio-political context, funding environment, external leadership, and values, needs and priorities of stakeholders and populations were addressed in over a third of the selected studies. Furthermore, the importance of process factors that to some extent overlap with those related to the outer setting, such as partnerships and engagement with stakeholders, effective communication with stakeholders and evaluations and data were discussed, especially in studies reporting on programs that were sustained for more than 5 years. As implementation and sustainability are on a continuum, groups designing and planning health programs should consider these outer setting and process factors that have been reported to impact health program sustainability.

Despite rapidly growing literature about healthcare program sustainability, there remains a lack of conceptual clarity in defining and assessing sustainability. Furthermore, frameworks such as the Consolidated Framework for Implementation Research [18] and RE-AIM (Reach, Effectiveness, Adoption, Implementation, Maintenance) [87] have been applied predominantly in the context of program implementation rather than sustainability. Applying these frameworks can be challenging when discerning the barriers/facilitators contributing to implementation as opposed to sustainability. Hall et al. [20] recommended the careful consideration of measures of determinants of sustainability that align with the construct of interest, such as objective and settings, to ensure robustness and relevancy of the program evaluation to sustainability. Moreover, it is important for future studies evaluating program sustainability to provide operational definitions of sustainability and clear evaluation timeframes as well as being explicit about theoretical frameworks to underpin their work.

## Conclusions

Strong leadership and stakeholder engagement, supportive organisational culture/climate, intervention fit with context and policy, intervention simplicity, adaptability, and fit with need and context, were all important factors in program sustainability. Adequate resourcing including ongoing availability of funding, training for staff, and low staff turnover, as well as constrained intervention costs, and alignment with organisational or broader policy or strategy were also strongly associated with program sustainability. Our review identifies the need for greater use of clear definitions of program sustainability and the application of validated frameworks in future research in this field. To that end, this review provided a working definition of each factor in the ISF to ensure consistency in defining barriers/facilitators associated with sustainability. Furthermore, a greater understanding of the factors associated with discontinuation of healthcare programs is needed, and this can only occur if negative outcomes are published to address the likely publication bias towards positive findings.

### Supplementary Information

**Additional file 1: Table S1.** PRISMA Checklist.

**Additional file 2: Table S2.** Search strategies used for systematic integrative review.

**Additional file 3: Table S3.** Quality assessment using Hawker’s Quality Assessment Tools. 

**Additional file 4: Table S4.** Barriers and facilitators using the ISFR2.

**Additional file 5: Table S5.** Long-term sustained programs (*n*= 29). 

## Data Availability

All data generated or analysed in this study are included in the published article.

## Abbreviations

ISF: Integrated Sustainability Framework
PRISMA: Preferred Reporting Items for Systematic Reviews and Meta-Analysis
RE-AIM: (Reach, Effectiveness, Adoption, Implementation, Maintenance)
