# Supplementary material for: Built to last? Barriers and facilitators of healthcare program sustainability: a systematic integrative review
Source: Implement Sci. 2023 Nov 13;18:62. doi: 10.1186/s13012-023-01315-x (PMC10641997; doi:10.1186/s13012-023-01315-x)
Supplement: Supplementary file 2 — Additional file 2: Table S2. Search strategies used for systematic integrative review. [file 13012_2023_1315_MOESM2_ESM.docx]

**Table S2.** Search strategies used for systematic integrative review

| **Medline** | |
| --- | --- |
| 1 | (Sustainab* or continuation or continual or institutionali* or resilien* or durab* or viab* or stability or stable or persist* or maintenance or routin*).ti,ab. |
| 2 | (Improve* or innovation or reform* or intervention or program* or strateg* or project or plan or change management).ti,ab. |
| 3 | Health Promotion/ or Program Evaluation/ or Organizational Innovation/ |
| 4 | 2 or 3 |
| 5 | "Delivery of health care"/ or "Delivery of health care, integrated"/ |
| 6 | 1 and 4 and 5 |
| 7 | limit 6 to (english language and humans and yr="2011 -Current" and (adaptive clinical trial or case reports or clinical study or clinical trial, all or clinical trial, phase i or clinical trial, phase ii or clinical trial, phase iii or clinical trial, phase iv or clinical trial or comparative study or controlled clinical trial or evaluation studies or meta analysis or observational study or pragmatic clinical trial or randomized controlled trial or "review" or systematic reviews)) |
| **CINAHL** | |
| 1 | (MH "Program Evaluation") OR TI(Sustainab* OR continuation OR continual OR institutionali* OR resilien* OR durab* OR viab* OR stability OR stable OR persist* OR maintenance OR routin*) OR AB(Sustainab* OR continuation OR continual OR institutionali* OR resilien* OR durab* OR viab* OR stability OR stable OR persist* OR maintenance OR routin*) |
| 2 | (MH "Hospital Programs") OR (MH "National Health Programs") OR (MH "Health Promotion") OR AB(Improve* OR innovation OR reform* OR intervention OR program* OR strateg* OR project OR plan OR change management) OR TI(Improve* OR innovation OR reform* OR intervention OR program* OR strateg* OR project OR plan OR change management) |
| 3 | (MH "Health Care Delivery") OR (MH "Health Care Delivery, Integrated") |
| 4 | 1 and 2 and 3  Limiters - Published Date: 20110101-20180331; English Language; Peer Reviewed; Publication Type: Case Study, Clinical Trial, Journal Article, Meta Analysis, Meta Synthesis, Nursing Interventions, Randomized Controlled Trial, Research, Review, Systematic Review |
| **EMBASE** | |
| 1 | (Sustainab* or continuation or continual or institutionali* or resilien* or durab* or viab* or stability or stable or persist* or maintenance or routin*).ti,ab. |
| 2 | exp program sustainability/ |
| 3 | (Improve* or innovation or reform* or intervention or program* or strateg* or project or plan or change management).ti,ab. |
| 4 | health program/ or health promotion/ or organization/ |
| 5 | health care delivery/ or integrated health care system/ |
| 6 | 1 or 2 |
| 7 | 3 or 4 |
| 8 | 5 and 6 and 7 |
| 9 | limit 8 to (human and english language and yr="2011 -Current" and (article or article in press or "review") and journal) |

**Table S2.** Continued

| **Scopus** | |
| --- | --- |
| 1 | TITLE ( sustainab* OR continuation OR continual OR institutionali* OR resilien* OR durab* OR viab* OR stability OR stable OR persist* OR maintenance OR routin* ) AND TITLE ( improve* OR innovation OR reform* OR intervention OR program* OR strateg* OR project OR plan OR change AND management ) AND ( LIMIT-TO ( SUBJAREA , "MEDI" ) OR LIMIT-TO ( SUBJAREA , "NURS" ) OR LIMIT-TO ( SUBJAREA , "HEAL" ) ) AND ( LIMIT-TO ( DOCTYPE , "ar" ) OR LIMIT-TO ( DOCTYPE , "re" ) OR LIMIT-TO ( DOCTYPE , "ip" ) ) AND ( LIMIT-TO ( LANGUAGE , "English" ) ) AND ( LIMIT-TO ( SRCTYPE , "j" ) ) AND ( LIMIT-TO ( PUBYEAR , 2018 ) OR LIMIT-TO ( PUBYEAR , 2017 ) OR LIMIT-TO ( PUBYEAR , 2016 ) OR LIMIT-TO ( PUBYEAR , 2015 ) OR LIMIT-TO ( PUBYEAR , 2014 ) OR LIMIT-TO ( PUBYEAR , 2013 ) OR LIMIT-TO ( PUBYEAR , 2012 ) OR LIMIT-TO ( PUBYEAR , 2011 ) ) |
| **Web of Science** | |
| 1 | TITLE: (( sustainab* OR continuation OR continual OR institutionali* OR resilien* OR durab* OR viab* OR stability OR stable OR persist* OR maintenance OR routin* )) AND TITLE: (( improve* OR innovation OR reform* OR intervention OR program* OR strateg* OR project OR plan OR change AND management )) AND TOPIC: (health)  Refined by: DOCUMENT TYPES: ( ARTICLE OR REVIEW )  LANGUAGES: ( ENGLISH )  Timespan: 2011-2018. Indexes: SCI-EXPANDED, SSCI. |
| **Emerald Management** | |
| 1 | ABSTRACT (Sustainab* OR continuation OR continual OR institutionali* OR resilien* OR durab* OR viab* OR stability OR stable OR persist* OR maintenance OR routin*)  AND  ABSTRACT (Improve* OR innovation OR reform* OR intervention OR program* OR strateg* OR project OR plan OR change management)  AND  KEYWORD (health care)  Publication Date: 01/01/2011 - 03/31/2018 |
