# Supplementary material for: Built to last? Barriers and facilitators of healthcare program sustainability: a systematic integrative review
Source: Implement Sci. 2023 Nov 13;18:62. doi: 10.1186/s13012-023-01315-x (PMC10641997; doi:10.1186/s13012-023-01315-x)
Supplement: Supplementary file 3 — Additional file 3: Table S3. Quality assessment using Hawker’s Quality Assessment Tools. [file 13012_2023_1315_MOESM3_ESM.docx]

**Table S3.** Quality assessment using Hawker’s Quality Assessment Tools

| Reference | Abstract and title | Introduction and aims | Method and data | Sampling | Data analysis | Ethics and bias | Results | Transfer-ability | Implications and usefulness | Overall rating | Quality assessment |
| --- | --- | --- | --- | --- | --- | --- | --- | --- | --- | --- | --- |
| Agarwal et al., 2012 | 4 | 4 | 4 | 3 | 4 | 2 | 4 | 1 | 3 | 29 | Moderate |
| Agarwal et al., 2016 | 4 | 3 | 4 | 2 | 4 | 2 | 4 | 2 | 2 | 27 | Moderate |
| Allegranzi et al., 2013 | 4 | 2 | 3 | 3 | 4 | 3 | 4 | 3 | 3 | 29 | Moderate |
| Ament et al., 2014 | 4 | 4 | 4 | 4 | 4 | 4 | 4 | 3 | 4 | 35 | High |
| Ament et al., 2017 | 4 | 4 | 4 | 3 | 4 | 3 | 4 | 3 | 4 | 33 | High |
| Assuncao et al., 2014 | 4 | 4 | 3 | 4 | 4 | 1 | 4 | 3 | 3 | 30 | High |
| Balfour et al., 2017 | 4 | 4 | 3 | 3 | 3 | 2 | 4 | 2 | 2 | 27 | Moderate |
| Benn et al., 2012 | 4 | 4 | 4 | 3 | 4 | 4 | 3 | 3 | 4 | 33 | High |
| Blanchet & James, 2014 | 4 | 4 | 4 | 4 | 3 | 2 | 4 | 4 | 4 | 33 | High |
| Block et al., 2018 | 4 | 4 | 4 | 3 | 4 | 4 | 4 | 3 | 3 | 33 | High |
| Bond et al.,2014 | 3 | 4 | 4 | 4 | 4 | 3 | 4 | 3 | 4 | 33 | High |
| Breckenridge-Sproat et al., 2017 | 4 | 4 | 4 | 3 | 4 | 2 | 4 | 3 | 4 | 32 | High |
| Brewster et al., 2015 | 3 | 2 | 3 | 4 | 4 | 2 | 4 | 4 | 3 | 29 | Moderate |
| Bridges et al., 2017 | 4 | 2 | 3 | 3 | 3 | 3 | 3 | 3 | 3 | 27 | Moderate |
| Burlew et al., 2014 | 4 | 4 | 3 | 3 | 3 | 1 | 3 | 3 | 3 | 27 | Moderate |
| Casati & Bjugn, 2012 | 4 | 4 | 4 | 4 | 4 | 3 | 4 | 4 | 2 | 33 | High |
| Chandani et al., 2017 | 4 | 4 | 4 | 4 | 4 | 1 | 4 | 4 | 3 | 32 | High |
| Cramm & Nieboer, 2014 | 4 | 4 | 4 | 3 | 4 | 2 | 4 | 2 | 2 | 29 | Moderate |
| Cresswell et al., 2012 | 4 | 4 | 4 | 3 | 4 | 4 | 4 | 3 | 4 | 34 | High |
| De Neve et al., 2017 | 4 | 3 | 4 | 4 | 4 | 3 | 4 | 4 | 4 | 34 | High |
| Fieldston et al., 2016 | 4 | 4 | 2 | 1 | 1 | 1 | 3 | 2 | 3 | 21 | Low |
| Fleiszer et al., 2016 | 4 | 4 | 4 | 4 | 4 | 3 | 4 | 4 | 4 | 35 | High |
| Ford et al., 2011 | 3 | 3 | 2 | 4 | 3 | 3 | 4 | 3 | 4 | 29 | Moderate |
| Gillissen et al., 2015 | 4 | 3 | 4 | 4 | 4 | 3 | 4 | 4 | 3 | 33 | High |
| Greenhalgh et al., 2012 | 4 | 4 | 4 | 3 | 4 | 4 | 4 | 3 | 4 | 34 | High |
| Hovlid et al., 2012 | 4 | 3 | 4 | 4 | 4 | 4 | 4 | 3 | 4 | 34 | High |
| Ilott et al., 2016 | 4 | 4 | 4 | 3 | 4 | 4 | 4 | 3 | 3 | 33 | High |

**Table S3.** Continued

| Reference | Abstract and title | Introduction and aims | Method and data | Sampling | Data analysis | Ethics and bias | Results | Transfer-ability | Implications and usefulness | Overall rating | Quality assessment |
| --- | --- | --- | --- | --- | --- | --- | --- | --- | --- | --- | --- |
| Jansink et al., 2013 | 3 | 3 | 4 | 2 | 4 | 4 | 3 | 2 | 2 | 27 | Moderate |
| Kanamori et al., 2015 | 4 | 4 | 3 | 3 | 4 | 4 | 4 | 3 | 4 | 33 | High |
| Kastner et al., 2017 | 4 | 4 | 4 | 3 | 4 | 4 | 4 | 2 | 3 | 32 | High |
| King et al., 2013 | 4 | 4 | 4 | 4 | 4 | 4 | 4 | 4 | 4 | 36 | High |
| Lean et al., 2015 | 4 | 4 | 4 | 3 | 4 | 4 | 4 | 3 | 3 | 33 | High |
| MacLean et al., 2013 | 3 | 4 | 4 | 3 | 4 | 3 | 4 | 3 | 3 | 31 | High |
| Magadzire et al., 2015 | 4 | 4 | 3 | 3 | 3 | 3 | 4 | 3 | 4 | 31 | High |
| Mahomed et al., 2016 | 3 | 4 | 4 | 3 | 4 | 4 | 4 | 3 | 3 | 32 | High |
| Makai et al., 2014 | 4 | 4 | 3 | 3 | 4 | 3 | 4 | 3 | 4 | 32 | High |
| Marten, 2017 | 3 | 3 | 3 | 2 | 3 | 1 | 3 | 2 | 3 | 23 | Low |
| Martin et al., 2016 | 4 | 3 | 3 | 3 | 4 | 4 | 3 | 3 | 3 | 30 | High |
| Mayer et al., 2011 | 4 | 3 | 3 | 3 | 3 | 1 | 4 | 3 | 4 | 28 | Moderate |
| Najafizada et al., 2017 | 4 | 3 | 4 | 4 | 3 | 4 | 4 | 4 | 2 | 32 | High |
| Neufeld & Case, 2013 | 4 | 4 | 3 | 4 | 3 | 3 | 4 | 3 | 3 | 31 | High |
| Ohinmaa et al., 2016 | 4 | 4 | 4 | 4 | 4 | 4 | 4 | 4 | 3 | 35 | High |
| Oliveira et al., 2017 | 4 | 3 | 3 | 2 | 3 | 3 | 4 | 2 | 4 | 28 | Moderate |
| Palinkas et al., 2011 | 3 | 4 | 4 | 3 | 4 | 4 | 4 | 2 | 4 | 32 | High |
| Parchman et al., 2013 | 4 | 4 | 3 | 4 | 4 | 4 | 4 | 3 | 4 | 34 | High |
| Paul & McDaniel, 2016 | 4 | 4 | 4 | 2 | 4 | 4 | 4 | 3 | 4 | 33 | High |
| Peterson et al., 2014 | 3 | 4 | 4 | 3 | 4 | 3 | 4 | 3 | 3 | 31 | High |
| Pomey et al., 2017 | 4 | 4 | 3 | 3 | 4 | 4 | 4 | 3 | 3 | 32 | High |
| Prashanth et al., 2014 | 4 | 4 | 4 | 4 | 3 | 2 | 4 | 4 | 3 | 32 | High |
| Qian et al., 2011 | 4 | 4 | 4 | 3 | 4 | 4 | 3 | 3 | 3 | 32 | High |
| Rakha et al., 2013 | 4 | 3 | 4 | 3 | 4 | 2 | 3 | 3 | 3 | 30 | High |
| Regagnin et al., 2016 | 4 | 3 | 3 | 3 | 3 | 3 | 4 | 3 | 3 | 29 | Moderate |

**Table S3.** Continued

| Reference | Abstract and title | Introduction and aims | Method and data | Sampling | Data analysis | Ethics and bias | Results | Transfer-ability | Implications and usefulness | Overall rating | Quality assessment |
| --- | --- | --- | --- | --- | --- | --- | --- | --- | --- | --- | --- |
| Rubin et al., 2011 | 3 | 4 | 3 | 3 | 2 | 1 | 3 | 3 | 4 | 26 | Moderate |
| Schuller et al., 2015 | 4 | 4 | 4 | 3 | 4 | 3 | 4 | 3 | 4 | 33 | High |
| Seppey et al., 2017 | 4 | 4 | 3 | 3 | 4 | 3 | 4 | 3 | 4 | 32 | High |
| Singh et al., 2017 | 4 | 4 | 4 | 3 | 4 | 3 | 4 | 3 | 4 | 33 | High |
| Sorensen et al., 2016 | 4 | 3 | 4 | 3 | 4 | 4 | 4 | 3 | 3 | 32 | High |
| Stirman et al., 2015 | 4 | 4 | 3 | 4 | 4 | 2 | 4 | 3 | 3 | 31 | High |
| Storm-Versloot et al., 2012 | 4 | 4 | 4 | 2 | 4 | 2 | 4 | 2 | 4 | 30 | High |
| Tjia et al., 2015 | 4 | 4 | 4 | 4 | 4 | 3 | 4 | 3 | 3 | 33 | High |
| Tomioka & Braun, 2015 | 4 | 4 | 3 | 2 | 3 | 3 | 4 | 2 | 4 | 27 | Moderate |
| van Rossum et al., 2016 | 4 | 3 | 3 | 4 | 4 | 1 | 4 | 4 | 4 | 31 | High |
| Weir et al., 2016 | 4 | 3 | 3 | 4 | 3 | 2 | 4 | 3 | 3 | 29 | Moderate |
| Wysham et al., 2014 | 4 | 4 | 4 | 3 | 2 | 2 | 4 | 3 | 3 | 29 | Moderate |
| Zakumumpa et al., 2016(a) | 4 | 4 | 4 | 4 | 4 | 4 | 4 | 4 | 3 | 35 | High |
| Zakumumpa et al., 2016(b) | 4 | 3 | 4 | 4 | 4 | 4 | 4 | 4 | 4 | 35 | High |
| Zakumumpa et al., 2017 | 4 | 4 | 4 | 4 | 4 | 3 | 4 | 4 | 4 | 35 | High |
| Aby, 2020 | 3 | 4 | 3 | 3 | 4 | 3 | 4 | 3 | 4 | 31 | High |
| Ali et al, 2021 | 4 | 4 | 3 | 2 | 2 | 3 | 4 | 3 | 3 | 28 | Moderate |
| Allchin et al, 2020 | 4 | 4 | 4 | 4 | 2 | 4 | 4 | 4 | 4 | 34 | High |
| Allen et al, 2020 | 4 | 4 | 2 | 2 | 2 | 2 | 4 | 4 | 4 | 28 | Moderate |
| Andrade et al., 2018 | 4 | 4 | 4 | 3 | 4 | 2 | 4 | 3 | 3 | 31 | High |
| Atif et al, 2019 | 4 | 4 | 4 | 4 | 4 | 3 | 4 | 4 | 4 | 35 | High |
| Avan et al, 2021 | 3 | 3 | 3 | 3 | 3 | 2 | 2 | 3 | 3 | 25 | Moderate |
| Azar et al, 2019 | 3 | 3 | 2 | 3 | 3 | 2 | 3 | 3 | 3 | 25 | Moderate |
| Beintner et al, 2020 | 4 | 4 | 3 | 3 | 3 | 3 | 3 | 3 | 4 | 30 | High |
| Bishop et al, 2020 | 4 | 4 | 4 | 4 | 4 | 3 | 4 | 4 | 4 | 35 | High |

**Table S3.** Continued

| Reference | Abstract and title | Introduction and aims | Method and data | Sampling | Data analysis | Ethics and bias | Results | Transfer-ability | Implications and usefulness | Overall rating | Quality assessment |
| --- | --- | --- | --- | --- | --- | --- | --- | --- | --- | --- | --- |
| Blervaque et al, 2021 | 4 | 4 | 4 | 3 | 3 | 3 | 3 | 2 | 4 | 30 | High |
| Budosan et al., 2020 | 4 | 3 | 3 | 3 | 3 | 1 | 4 | 3 | 4 | 28 | Moderate |
| Butow et al, 2019 | 4 | 3 | 3 | 4 | 3 | 3 | 4 | 3 | 4 | 31 | High |
| Chiliza et al, 2021 | 4 | 4 | 4 | 3 | 3 | 4 | 3 | 3 | 4 | 32 | High |
| Cranley et al, 2018 | 4 | 4 | 4 | 4 | 4 | 3 | 4 | 3 | 4 | 34 | High |
| Dharmayat et al, 2019 | 4 | 4 | 3 | 3 | 4 | 4 | 3 | 3 | 4 | 32 | High |
| Duggleby et al, 2020 | 4 | 4 | 3 | 2 | 4 | 2 | 4 | 3 | 4 | 30 | High |
| Fossey et al, 2019 | 3 | 4 | 4 | 4 | 3 | 3 | 3 | 3 | 3 | 30 | High |
| Fox et al, 2018 | 4 | 4 | 4 | 3 | 4 | 2 | 3 | 3 | 4 | 31 | High |
| Gramlich et al, 2020 | 4 | 3 | 4 | 3 | 4 | 3 | 4 | 2 | 2 | 29 | Moderate |
| Gray et al, 2018 | 4 | 3 | 3 | 2 | 2 | 2 | 4 | 2 | 3 | 25 | High |
| Gunaratnam et al, 2019 | 3 | 3 | 3 | 4 | 3 | 4 | 4 | 4 | 3 | 31 | High |
| Healey et al, 2019 | 4 | 3 | 3 | 2 | 2 | 3 | 4 | 2 | 3 | 26 | Moderate |
| Hoben et al, 2021 | 4 | 4 | 4 | 4 | 4 | 4 | 4 | 3 | 3 | 34 | High |
| Huang et al, 2018 | 4 | 4 | 3 | 2 | 3 | 3 | 3 | 2 | 3 | 27 | Moderate |
| Jabeen et al, 2022 | 4 | 4 | 4 | 4 | 3 | 3 | 4 | 3 | 3 | 32 | High |
| Kavanagh et al, 2020 | 4 | 4 | 4 | 3 | 4 | 2 | 4 | 3 | 3 | 31 | High |
| Kenu et al, 2017 | 4 | 3 | 3 | 3 | 3 | 4 | 4 | 3 | 3 | 30 | High |
| Lachman et al, 2021 | 4 | 2 | 3 | 3 | 3 | 2 | 4 | 4 | 4 | 29 | Moderate |
| Laur et al, 2018 | 4 | 4 | 4 | 4 | 4 | 3 | 4 | 4 | 3 | 34 | High |
| Louch et al, 2019 | 4 | 4 | 3 | 2 | 4 | 2 | 4 | 3 | 3 | 29 | Moderate |
| Morgan et al, 2019 | 4 | 3 | 4 | 3 | 4 | 3 | 4 | 4 | 4 | 33 | High |
| Nagelkerk et al, 2021 | 3 | 3 | 3 | 2 | 3 | 4 | 3 | 4 | 3 | 28 | Moderate |
| Nahimana et al, 2021 | 4 | 4 | 3 | 3 | 3 | 3 | 4 | 3 | 3 | 30 | High |
| Newbould et al, 2021 | 4 | 4 | 3 | 3 | 3 | 3 | 4 | 3 | 3 | 30 | High |
| Olumide et al, 2020 | 4 | 3 | 3 | 3 | 4 | 4 | 4 | 3 | 3 | 31 | High |
| Pekkala et al, 2020 | 4 | 4 | 3 | 2 | 2 | 3 | 4 | 2 | 3 | 27 | Moderate |
| Pennington et al, 2019 | 2 | 4 | 2 | 2 | 1 | 1 | 2 | 2 | 2 | 18 | Low |

**Table S3.** Continued

| Reference | Abstract and title | Introduction and aims | Method and data | Sampling | Data analysis | Ethics and bias | Results | Transfer-ability | Implications and usefulness | Overall rating | Quality assessment |
| --- | --- | --- | --- | --- | --- | --- | --- | --- | --- | --- | --- |
| Peracca et al, 2021 | 4 | 4 | 3 | 4 | 4 | 1 | 4 | 4 | 1 | 29 | Moderate |
| Perialathan et al, 2021 | 4 | 4 | 3 | 3 | 2 | 4 | 4 | 3 | 3 | 30 | High |
| Popowich et al, 2020 | 4 | 4 | 4 | 4 | 4 | 4 | 4 | 4 | 4 | 36 | High |
| Rogers et al, 2018 | 4 | 3 | 3 | 2 | 4 | 2 | 3 | 2 | 3 | 26 | Moderate |
| Ryba et al, 2021 | 4 | 3 | 3 | 3 | 4 | 3 | 4 | 4 | 4 | 32 | High |
| Seaman et al, 2022 | 4 |  | 4 | 4 | 4 | 3 | 3 | 3 | 3 | 28 | Moderate |
| Song et al, 2022 | 4 | 4 | 4 | 4 | 4 | 4 | 4 | 4 | 4 | 36 | High |
| Tanzi et al, 2020 | 3 | 2 | 3 | 3 | 4 | 3 | 3 | 2 | 3 | 26 | Moderate |
| Taxter et al, 2019 | 4 | 4 | 4 | 4 | 4 | 2 | 4 | 3 | 3 | 32 | High |
| Tejedor-Sojo et al, 2019 | 3 | 3 | 3 | 3 | 3 | 2 | 4 | 3 | 3 | 27 | Moderate |
| Thota et al, 2020 | 3 | 2 | 3 | 2 | 1 | 1 | 3 | 2 | 4 | 21 | Low |
| Urquhart et al, 2021 | 4 | 4 | 4 | 3 | 4 | 3 | 4 | 4 | 4 | 34 | High |
| Verma et al, 2018 | 3 | 4 | 4 | 3 | 4 | 3 | 4 | 3 | 4 | 32 | High |
| Vidgen et al, 2018 | 4 | 4 | 3 | 3 | 4 | 4 | 4 | 3 | 4 | 33 | High |
| Vinson et al, 2022 | 4 | 3 | 4 | 3 | 4 | 4 | 4 | 4 | 4 | 34 | High |
| Waiswa et al, 2021 | 4 | 4 | 2 | 2 | 1 | 1 | 4 | 3 | 4 | 25 | Moderate |
| Williams et al, 2019 | 4 | 4 | 4 | 4 | 4 | 2 | 4 | 2 | 3 | 31 | High |
| Zafar et al, 2019 | 3 | 2 | 4 | 1 | 2 | 1 | 4 | 2 | 3 | 22 | Low |
| Zakumumpa et al, 2018 | 4 | 4 | 4 | 4 | 4 | 3 | 4 | 4 | 4 | 35 | High |
