# Supplementary material for: Built to last? Barriers and facilitators of healthcare program sustainability: a systematic integrative review
Source: Implement Sci. 2023 Nov 13;18:62. doi: 10.1186/s13012-023-01315-x (PMC10641997; doi:10.1186/s13012-023-01315-x)
Supplement: Supplementary file 5 — Additional file 5: Table S5. Long-term sustained programs (n= 29). [file 13012_2023_1315_MOESM5_ESM.docx]

**Table S4.** Long-term sustained programs (n= 29)

| **Study details** | **Setting** | **Program** | **Evaluation timeframe** | **Details of sustainability** | **Facilitators of sustainability** |
| --- | --- | --- | --- | --- | --- |
| Allen KL, Mountford V, Brown A, Richards K, Grant N, Austin A, et al. First episode rapid early intervention for eating disorders (FREED): From research to routine clinical practice. Early Intervention in Psychiatry. 2020;14(5):625-30. | Eating disorder services | The First Episode Rapid Early Intervention for Eating Disorders (FREED) care package | 5 years (including 2 years of scaling up) | FREED is now embedded in  services without external financial support (i.e., research  grant support) and we have evidence of sustained improvements after the introduction of FREED. | - Buy-in locally by creating a shared sense of ownership - Continued support by ongoing supervision and FREED network - A train-the-trainer approach and sharing successes at a service and regional level - Joint collaborators with partner sites to ensure sustained scaling and adaptation - Engaging different target audiences in varied and tailored ways - Positive attitude from the FREED Champion |
| Ament SM, Gillissen F, Maessen JM, et al. Sustainability of short stay after breast cancer surgery in early adopter hospitals. Breast. 2014;23(4):429-34. | Four hospitals, breast cancer surgery services (the Netherlands) | Short-stay program in breast cancer surgery care | 5 years | The compliance with recommendations to facilitate short stay after breast cancer surgery increased from 65% after implementation to 78% five years post-implementation. | - Normalisation of short-stay breast cancer surgical protocols - The organisational culture in early adopter hospitals may have facilitated the sustained results - Adaptability of the new program and healthcare workers’ compliance with the key program recommendations |
| Ament SM, Gillissen F, Moser A, Maessen JM, Dirksen CD, von Meyenfeldt MF, van der Weijden T. Factors associated with sustainability of 2 quality improvement programs after achieving early implementation success: A qualitative case study. J Eval Clin Pract. 2017;23(6):1135-43. | 14 hospitals from the primary implementation projects | Two implementation cases, Enhanced Recovery After Surgery (ERAS) and Short-stay program in breast cancer surgery (SSP), executed by the Maastricht University Medical Centre | 3 to 6 years | Key factors associated with sustainability of 2 hospital-based multidisciplinary programs, in hospitals where early postimplementation success was achieved, 3 to 6 years after the completion of successful implementation. | - Modification and adaptability of the program to local and patient needs - Planning surgery in the early morning - Short formal and informal communication lines within the multidisciplinary team - Benefits to patients and client-centredness - The existence of external policies and incentives - Trust and belief in the program |
| Andrade, M. V., et al. Brazil's Family Health Strategy: factors associated with programme uptake and coverage expansion over 15 years (1998-2012). Health Policy Plan. 2018;33(3): 368-380. | 5419 municipalities in Brazil | The Family Health Strategy (FHS) | 15 years | The FHS coverage increased significantly over time from 50% in 1998 to 95% in 2012. | - Political party alignment of Mayors and Governors favoured both the adoption and the expansion of the programme - Regional characteristics (e.g., the size of the municipality, population size) |

| **Study details** | **Setting** | **Program** | **Evaluation timeframe** | **Details of sustainability** | **Facilitators of sustainability** |
| --- | --- | --- | --- | --- | --- |
| Atif N, Bibi A, Nisar A, Zulfiqar S, Ahmed I, Lemasters K, et al. Delivering maternal mental health through peer volunteers: A 5-year report. International Journal of Mental Health Systems. 2019;13. | Peri and post-natal support for women in the community with depression in Kallar Syeddan | The Thinking Healthy Programme (THP) | 5 years | Majority (70%) of the peer volunteers were retained over the 5-year period with achieving satisfactory competence. | - Sustained motivation included altruistic aspirations - Enhanced social standing in the community - Personal benefits to volunteers’ own mental health - Opportunity for other avenues of employment |
| Azar KMJ, Nasrallah C, Szwerinski NK, Petersen JJ, Halley MC, Greenwood D, et al. Implementation of a group-based diabetes prevention program within a healthcare delivery system. BMC Health Services Research. 2019;19:694. | Northern California multi-speciality healthcare | Group-based Diabetes Prevention Programs (DPP) | 6 years | A dynamic and important tension between the desire to retain fidelity to the original evidence-based program and the need to tailor the program to meet the local needs of the organisation, distinct patient populations, and the clinical context. | - Informing and reminding physicians about the program increased patients’ referral to the program - Prompt pay discount or allowing for monthly or per-session payment - Implementing a rolling enrollment system - Rescheduling sessions to fill classes and referring patients to other nearby sites in case of lack of available spaces |
| Blervaque L, Prefaut C, Forthin H, Maffre F, Bourrelier M, Heraud N, et al. Efficacy of a long-term pulmonary rehabilitation maintenance program for COPD patients in a real-life setting: a 5-year cohort study. Respiratory Research. 2021;22(1). | 144 chronic obstructive pulmonary disease (COPD) | Pulmonary rehabilitation maintenance program for COPD patients | 5 years | Maintenance program patients showed significant pulmonary rehabilitation (PR) benefits at 4 years for 6-minute walking distance (6MWD) and health-related quality of life using VQ11 and 5 years for dyspnea (MRC). | - Ongoing support from healthcare professionals - The self-help organisation and discussion groups provided peer interactions, self-monitoring and routines - Continuous psychological and social support from the self-help association |
| Bond GR, Drake RE, McHugo GJ, Peterson AE, Jones AM, Williams J. Long-term sustainability of evidence-based practices in community mental health agencies. Adm Policy Ment Health.41(2):228-236. | 49 community mental health agencies | The National Implementing Evidence-Based Practices Project | 6 years | 47% of the 49 sites were sustained over a 6-year period after the end of the 2-year implementation phase. | - Financial, prioritisation, leadership, workforce, workflow, reinforcement, population - Regular fidelity and outcome monitoring - Training for both new hires and experienced staff - Regular weekly supervision - Appropriate adequate funding and systematic monitoring |

| **Study details** | **Setting** | **Program** | **Evaluation timeframe** | **Details of sustainability** | **Facilitators of sustainability** |
| --- | --- | --- | --- | --- | --- |
| Burlew R, Puckett A, Bailey R, Caffrey M, Brantley S. Assessing the relevance, efficiency, and sustainability of HIV/AIDS in-service training in Nigeria. Human Resources for Health. 2014;12:20. | 39 PEPFAR-funded implementing partners offering in service training (IST) | the USAID-funded CapacityPlus project conducted an assessment of PEPFAR-funded IST in Nigeria | 5.5 Years | Sustainability outcomes: "Financial sustainability planning for in-service training (IST)" and "Sustainability - human resources and organizational capacity" | - Advocacy to the State Ministry of health (SMOH) and Federal Ministry of health (FMOH) for a budgetary allocation - Involving the SMOH and FMOH in training - Aligning the IST with existing national curricula and standards - The capacity of local trainers was developed via training-of-trainers. |
| Casati B, Bjugn R. Structured electronic template for histopathology reporting on colorectal carcinoma resections: Five-year follow-up shows sustainable long-term quality improvement. Archives of Pathology & Laboratory Medicine. 2012;136(6):652-6. | One pathology department implementing a national initiative (Norway) | A national electronic template for histopathology reporting of colorectal carcinoma resections implemented into daily routine practice | 5 years | The template was used in 91.8% of cases (n=1186) and had significantly improved upon the reporting of parameters at 5 years post-implementation. | - Template clarity and simplicity - Health leaders as champions - Culture of support for the template from staff members and peers - Ongoing National funding - Change in individual long-term behavior to refer to the national electronic template and include key parameters when writing reports |
| Gillissen F, Ament SMC, Maessen JMC, et al. Sustainability of an Enhanced recovery after surgery program (ERAS) in colonic surgery. World Journal of Surgery.39(2):526-533. | 10 hospitals involved in the Enhanced Recovery After Surgery (ERAS) program for colonic surgery | the Enhanced Recovery After Surgery (ERAS) program | 3 to 5 years | Despite a large variation, the ERAS program was sustained reasonably well in the 10 selected hospitals even though there was a large variation among hospitals. | - The awareness of being evaluated - Protocol adherence (though to what extent is unclear, as hospitals with lower adherence rates had low LOS and vice versa) |
| Kavanagh AY, O'Brien LJ, Maloney SR, Osadnik CR. Barriers and facilitators to adopting functional maintenance initiatives for acutely hospitalised older adults. Disability & Rehabilitation. 2020;42(26):3808-15. | Five international and four Victorian FMIs | Functional maintenance initiatives (FMIs) | Up to 23 years after the start year | Successful adoption and sustainability of functional maintenance initiatives for acutely hospitalised older adults are influenced by a range of “non-clinical” factors, such as the ongoing effectiveness of change drivers. | - Support from change drivers/champions (staff) - Having a highly placed stakeholder as the initiative’s champion - Integrating interventions and communication process into usual care - Accompanying training strategy - Evaluation strategy and monitoring - Serves institutional priorities |

| **Study details** | **Setting** | **Program** | **Evaluation timeframe** | **Details of sustainability** | **Facilitators of sustainability** |
| --- | --- | --- | --- | --- | --- |
| Kenu A, Kenu E, Bandoh DA, Aikins M. Factors that promote and sustain the use of traditional, complementary and integrative medicine services at LEKMA hospital, Ghana, 2017: An observational study. BMC Complementary Medicine and Therapies. 2021;21. | Ledzokuku Krowo Municipal Assembly (LEKMA) hospital, a public facility at the district level | Complementary and alternative medicine therapy services | 6 years | The high usage of TCIM among users of TCIM at LEKMA indicated that it was sustainable from the clients’ perspective. However, it was partially sustainable from the health service providers’ perspective. | - The services were being monitored (e.g., submitting regular reports) - Funding supported by Chinese government - Training opportunity in China - Political support from local authorities - Ghana health services advocated for TCIM services |
| Magadzire BP, Marchal B, Ward K. Improving access to medicines through centralised dispensing in the public sector: A case study of the chronic dispensing unit in the Western Cape Province, South Africa. BMC Health Services Research. 2015;15(1):513. | The Western Cape Department of Health (WCDoH) (South Africa) | Chronic Dispensing Unit (CDU) | 10 years | Started with eight centres in 2005 and reached 216 centres by 2015. | - Leadership and support from funders - Engagement with clinical staff involved in developing the program - Partnership between the WCDoH and the contractor (UTI Pharma) - Adaptability and flexibility between involved stakeholders, e.g., adapting to policy changes - Policies alignment at multiple organisational levels, and with the implementation team (health personnel and the contractor) - Technological advancements (e.g., machines allowing the picking, packaging and labeling of medicines) that increased scope of program - Funding for five years from government and contractors through political alignment |
| Ohinmaa A, Zheng Y, Jeerakathil T, et al. Trends and regional variation in hospital mortality, length of stay and cost in hospital of ischemic stroke patients in Alberta accompanying the provincial reorganization of stroke care. J Stroke Cerebrovasc Dis. 2016;25(12):2844-2850. | Acute care hospitals in Alberta | Alberta Provincial Stroke Strategy (APSS) | 6 years | The APSS was associated with a reduction in mortality for ischemic stroke, a decrease in the cost of admission for all stroke types, and a reduction in variability between sites admitting stroke patients. | - Lower cost of stroke care - Linkage of the DAD registry data - Targeted patients in the ALC designation - Improvements in inpatient rehabilitation capacity |

| **Study details** | **Setting** | **Program** | **Evaluation timeframe** | **Details of sustainability** | **Facilitators of sustainability** |
| --- | --- | --- | --- | --- | --- |
| Oliveira SRA, Medina MG, Figueiro AC, Potvin L. Strategic factors for the sustainability of a health intervention at municipal level of Brazil. Cadernos de Saude Publica. 2017;33(7):e00063516. | Primary care—over 39,000 family health units [FHUs]) (Brazil) | The Family Health Program (FHP) implemented in 1994 as national policy | 12 years | Six critical events that allowed the program to evolve and change in response to its context, thus facilitating long-term sustainability. | - The intervention was compatible with the government’s vision, allowing it to withstand policy and political changes occurring in local health management - The change of municipal management by mobilising champions to rebuild alliances with other sectors for collaboration - The involvement of staff as decision makers and negotiators to maintain the stability of the intervention - Ongoing monitoring and adaptation of the program in response to critical events that occurred in the 11 years since the program’s inception, e.g., staff turnover and navigating different council approaches to healthcare |
| Paul DL, McDaniel RR. Facilitating telemedicine project sustainability in medically underserved areas: A healthcare provider participant perspective. BMC Health Services Research. 2016;16:148. | 14 teleconsultation projects that were part of two health sciences centre (HSC) based telemedicine networks | Teleconsultation projects in medically underserved areas or populations | 10 years | The fundamental purpose of teleconsultation projects needs to be re-conceptualised to increase sustainability. | - Improve healthcare delivery at remote sites - Less dedicated time to teleconsultation sessions compared to face-to-face - Active participation and collaboration across the healthcare providers involved - Reliable and suitable technology for the demand learning - Continuous learning process - Designing the consultation process as a learning process |
| Peterson AE, Bond GR, Drake RE, McHugo GJ, Jones AM, Williams JR. Predicting the long-term sustainability of evidence-based practices in mental health care: An 8-year longitudinal analysis. The Journal of Behavioural Health Services & Research. 2014;41(3):337-346. | 49 sites that implemented evidence-based practices (EBPs) | National Implementing Evidence-Based Practices Project | 8 years | A total of 39 sites survived at year 4 and 23 survived at year 8, suggesting an annual continuation rate of roughly 89%. | - Collecting and monitoring - The important role of policymakers - Continuous support from supervisors - Regular training for new hires and current staff - Field mentoring - Sufficient funding |

| **Study details** | **Setting** | **Program** | **Evaluation timeframe** | **Details of sustainability** | **Facilitators of sustainability** |
| --- | --- | --- | --- | --- | --- |
| Regagnin DA, Alves DSD, Cavalheiro AM, et al. Sustainability of a program for continuous reduction of catheter-associated urinary tract infection. American Journal of Infection Control. 2016;44(6):642-646. | 2 medical-surgical ICUs with 48 beds and 3 step-down units with 95 beds in a private tertiary care hospital in Sao Paulo. | Educational intervention to reduce catheter-associated urinary tract infection (CAUTI) | 6 years | Comparisons of CAUTI rates in the 3 study phases, both in the ICUs and SDUs, showed significant reductions both between the 3 periods and in all possible combinations of analysis phases (all p < .001). It requires a multidisciplinary team with different strategies that require continuous monitoring to sustain the reductions of CAUTI rates. | - Committed and engaged team members in the UC team - Sharing the results of the nursing staff related to the CAUTI rates - A cultural change to develop a greater awareness - Daily assessments of all the beds in the ICUs and SDUs with results monitored by a senior nurse and shared with all nursing staff - Didactic training - Collaboration with medical teams on early removal catheters |
| Rogers EA, Manser ST, Cleary J, Joseph AM, Harwood EM, Call KT. Integrating community health workers into medical homes. Annals of Family Medicine. 2018;16:14-20. | Nine clinics (five with community health workers, four without) in Minnesota | State PCMH certification  is a redesign of care delivery and payment  reform | 5 years | Integration requires a champion with awareness of potential CHW value, a clinic culture of innovation with pilot resources, perception of patient needs spanning medical and nonmedical, determinants of health, and finding sustainable reimbursement strategies. | - - The influence of the positive image of CHW champions   - The influence of care team redesign mandates required for PCMH certification   - Acknowledging patients’ nonmedical needs (e.g., psychosocial, cultural, socioeconomic nee   - The financial sustainability potential of CHWs - Dedicated role to care coordination |
| Rubin FH, Neal K, Fenlon K, Hassan S, Inouye SK. Sustainability and scalability of the hospital elder life program at a community hospital. J Am Geriatr Soc. 2011;59(2):359-365. | Shadyside, a 500-bed community teaching hospital in Pittsburgh | The Hospital Elder Life Program (HELP) | 7 years | The multiplicative expansion of the program during this timeframe attests to the scalability and generalisability of the HELP interventions. | - Institutional commitment and administrative buy-in - Keeping the program visible through awards and commendations - Ongoing demonstrations of the clinical and financial benefits of the program - Strong clinical leadership - Ability to change organisational culture to support the innovation - Providing credible supportive data and developing an infrastructure supportive to the innovation - High fidelity to the original model while making local adaptations necessary to program survival |

| **Study details** | **Setting** | **Program** | **Evaluation timeframe** | **Details of sustainability** | **Facilitators of sustainability** |
| --- | --- | --- | --- | --- | --- |
| Schuller KA, Kash BA, Gamm LD. Studer Group's evidence-based leadership initiatives. Journal of Health Organization and Management. 2015;29(6):684-700. | Two large health systems: Long Hospital-over 500 beds, a metropolitan, teaching hospital. Short health system - metropolitan hospitals with well over 500 beds | Studer Groups' Evidence-Based Leadership (EBL) | 3 and 7 years | The three themes associated with success and sustainability of the EBL initiative related to leadership, culture, and organisational processes. | - Sense of accountability and creation of a positive work environment - Staff and organisational buy-in - The use of rewards and recognition - Engaged and committed leaders - Strong leadership - Charismatic and passionate leaders - Training and external Studer Groups coaches - Implementation strategies |
| Stirman S, Matza A, Gamarra J, et al. System-level influences on the sustainability of a cognitive therapy program in a community behavioral health network. Psychiatric Services. 2015;66(7):734-742 | A community-based mental health system with over 300 provider agencies providing mental health and substance abuse services. | A community-based partnership to implement cognitive therapy (CT) for depression and suicide prevention and for disorders that commonly co-occur with depression | 6 years | A number of interconnected and interacting influences on sustainability at the system level, such as alignment with emerging sociopolitical influences and system and organisational priorities; program-level adaptation and evolution; intervention flexibility. | - Processes and policies that clarified the roles and responsibilities - Strong collaborative relationship between stakeholders - Alignment with emerging sociopolitical influences and system and organisational priorities - Program-level adaptation and evolution; Intervention fit and flexibility - Planning and support: technical assistance and financial - Evaluation and perceived benefit. |
| Storm-Versloot MN, Knops AM, Ubbink DT, Goossens A, Legemate DA, Vermeulen H. Long-term adherence to a local guideline on postoperative body temperature measurement: mixed methods analysis. J Eval Clin Pract. 2012;18(4):841-847. | One surgical department in a 1000-bed university hospital | Assessment of long-term guideline adherence on postoperative body temperature (BTM) | 7 years | An initially applied successful multifaceted implementation strategy does not guarantee long-term adherence to an evidence-based guideline | - Trust in in the scientific basis of the guideline - Preventing superfluous diagnostic tests - Stimulating the development of clinical judgement - Confidence in personal clinical judgement for identifying a postoperative infection - Staff support in using the guideline - Nurses correct each other when inappropriate measurements were taken |

| **Study details** | **Setting** | **Program** | **Evaluation timeframe** | **Details of sustainability** | **Facilitators of sustainability** |
| --- | --- | --- | --- | --- | --- |
| Weir SS, Page C, Newton WP. Continuity and access in an academic family medicine center. Family Medicine. 2016;48(2):100-107. | The University of North Carolina (UNC) Family Medicine Center in a large academic teaching practice | Breakthrough Series Collaborative focused on improving appointment access and efficiency and sponsored by the National Institute for Child Healthcare Quality (NICHQ) | 5 years | The improvements in usual provider continuity and time to third available appointment were sustained over five years. | - Promoting a culture of transparent data reporting - Celebrating successes whenever possible - Posting monthly usual team continuity results - Balance supply and demand - Simplifying appointment types and durations - Anticipating backlog from forming with “individual contingency plans” - New scheduling systems - Dedicated scheduling coordinator |
| Zakumumpa H, Bennett S, Ssengooba F. Accounting for variations in ART program sustainability outcomes in health facilities in Uganda: A comparative case study analysis. BMC Health Services Research. 2016;16:1-3. | Six health facilities receiving donor support for antiretroviral therapy (ART): High Sustainers (2), Low Sustainers (2) and non-Sustainers (2). | National ART scale-up program | 11 years (data collected 6 years after funding ended) | The ART program sustainability was embedded in a complex system involving dynamic interactions between internal (program champion, staffing strength, M &E systems, goal clarity) and external drivers (donors, ARVs supply chain, patient demand). | **High Sustainers:**   - Higher staffing and patient volumes - A broader ‘menu’ of ART services - More stable program leadership compared to the other cases - Multiple funding streams - Robust ART program evaluation systems - Internal and external program champions |
| Zakumumpa H, Taiwo MO, Muganzi A, Ssengooba F. Human resources for health strategies adopted by providers in resource-limited settings to sustain long-term delivery of ART: A mixed-methods study from Uganda. Human Resources for Health. 2016;14:1-1. | 195 health facilities in Uganda which were accredited to provide ART | Uganda implemented an emergency national antiretroviral therapy (ART) scale-up program between 2004 and 2009 with PEPFAR and Global Fund support. | 10 years (data collected 5 years after funding ended) | Facility-level strategies for responding to human resources for health constraints are feasible and can contribute viable bottom-up solutions to efforts to increase country ownership of HIV programs in countries dependent on external donor support. | - Providing monetary and non-monetary incentives to health workers on busy ART clinic days - Workload reduction through spacing ART clinic appointments - Adopting training workshops in ART management as a motivation strategy for health workers - Adopting non-physician-centred staffing models - Devising ART program leadership styles that enhanced health worker commitment |

| **Study details** | **Setting** | **Program** | **Evaluation timeframe** | **Details of sustainability** | **Facilitators of sustainability** |
| --- | --- | --- | --- | --- | --- |
| Zakumumpa H, Bennett S, Ssengooba F. Modifications to ART service delivery models by health facilities in Uganda in promotion of intervention sustainability: A mixed methods study. Implementation Science. 2017;12(1):1-4. | 195 health facilities in Uganda which were accredited to provide ART | National ART scale-up program | 10 years (data collected 5 years after funding ended) | Health facilities made several modifications ART interventions to improve fit with their resource-constrained settings thereby promoting long-term sustainability. | - Reducing the frequency of clinic appointments and pharmacy-only refill programs - Modifying the guidelines based on patients’ conditions - Home-based care programs were introduced to reduce provider ART delivery costs - Task shifting to non-physician cadre was reported in 181 (93%) of the health facilities - Two health facilities focused on patients living nearer the health facilities to align with targets set by external donors. |
| Zakumumpa H, Kwiringira J, Rujumba J, Ssengooba F. Assessing the level of institutionalization of donor-funded anti-retroviral therapy (ART) programs in health facilities in Uganda: Implications for program sustainability. Global Health Action. 2018;11(1):1523302. | Four health facilities were purposively selected, which two sites with the highest institutionalisation scores and two the lowest institutionalisation scores. (Uganda) | Anti-retroviral therapy (ART) scale-up | Implementation history of between 8 and 11 years (sites with the highest scores) | Two sites with the highest institutionalisation had successfully transitioned to a more ‘permanent’ state in ART service delivery whereas two sites with the lowest scores were in ‘pilot’ mode. | - The availability of written procedures and manuals for ART service delivery and organisational strategic plans - Adaptations to program models when needed - Formally appointed roles - The presence of program champion - Service support from other departments - ART program was embedded in daily organisational routines - Deliberate strategies for training and retention of ART-proficient staff - Salary support for key staff in the ART clinic |
